# A rare case report of enteric fever causing gallbladder perforation

**DOI:** 10.1016/j.ijscr.2021.106553

**Published:** 2021-11-02

**Authors:** Tika Ram Bhandari, Sarfaraz Alam Khan, Jiuneshwar Lal Jha, Jayant Kumar Sah

**Affiliations:** a Department of General Surgery, People's Dental College and Hospital, Kathmandu, Nepal; b Department of General Surgery, Institute of Medicine, Tribhuvan University Teaching Hospital, Kathmandu, Nepal

**Keywords:** Enteric fever, Gallbladder perforation, Adult patient, Case report

## Abstract

**Introduction and importance:**

Enteric fever is one of the major public health problems mainly in developing countries. Gallbladder perforation is very unusual. Enteric fever rarely causes gallbladder perforation. We report a case of gallbladder perforation due to enteric fever in an adult patient.

**Case presentation:**

A 50-year-old female without any medical illness presented with a history of intermittent fever for two weeks and three days duration of severe abdominal pain. Upper abdominal tenderness and guarding were found in the abdominal examination. Ultrasonography showed thickening of the gallbladder wall and pericholecystic fluid collection. Magnetic resonance cholangiopancreatography revealed a distended gallbladder with sludge, diffuse wall thickening, and contained perforation with a mild amount of free fluid seen in the abdomen. With the diagnosis of type II gallbladder perforation, percutaneous ultrasonography-guided drainage was done. The culture of bile revealed positivity for *Salmonella Typhi*. Intra-venous antibiotic (ceftriaxone and gentamicin) was administered for 14 days. Four weeks later, cholecystectomy with peritoneal lavage was done. She was discharged on the 8th postoperative day.

**Clinical discussion:**

Preoperative diagnosing of gallbladder perforation is challenging. The accurate treatment and precise timing of the surgery remain important. In most cases, cholecystectomy and abdominal lavage are adequate to treat gallbladder perforation.

**Conclusions:**

Gallbladder perforation is a life-threatening surgical problem. The clinician should have a high index of awareness about this unusual surgical entity due to enteric fever and early diagnosis with prompt surgical intervention is necessary to improve patient outcomes.

## Highlights

• Enteric fever is one of the major public health problems mainly in developing countries.
• Gallbladder perforation is a very uncommon complication of enteric fever.
• Awareness of this rare entity due to enteric fever is important for prompt timely management for better patient outcomes.

## 1 Introduction and importance

Gallbladder perforation is a rare surgical emergency usually missed as a differential diagnosis of acute abdomen. Though Enteric fever is one of the major public health problems particularly in developing countries, gallbladder perforation in association with enteric fever is only about 3%–10% [1]. The causative agents for enteric fever are *Salmonella typhi*, *S. paratyphi* A, and less commonly *S. para*-typhi B and C that multiply in the bile and are concentrated in the gallbladder. Moreover, it can invade gallbladder epithelial cells, causing gallbladder wall perforation [2]. Preoperative diagnosis is challenging; delayed diagnosis and treatment can lead to high morbidity and mortality [3]. Therefore, early diagnosis and early surgical intervention can be a lifesaving approach in such cases. Here, we report the case of a 50-year old female with gallbladder perforation, who presented with an acute abdomen and improved after surgical intervention. This case was managed by team of consultant general surgeon in academic teaching hospital.

## 2 Method

We report this case in line with the updated consensus-based surgical case report (SCARE) guidelines [4].

## 3 Case presentation

**Fig. 1:**
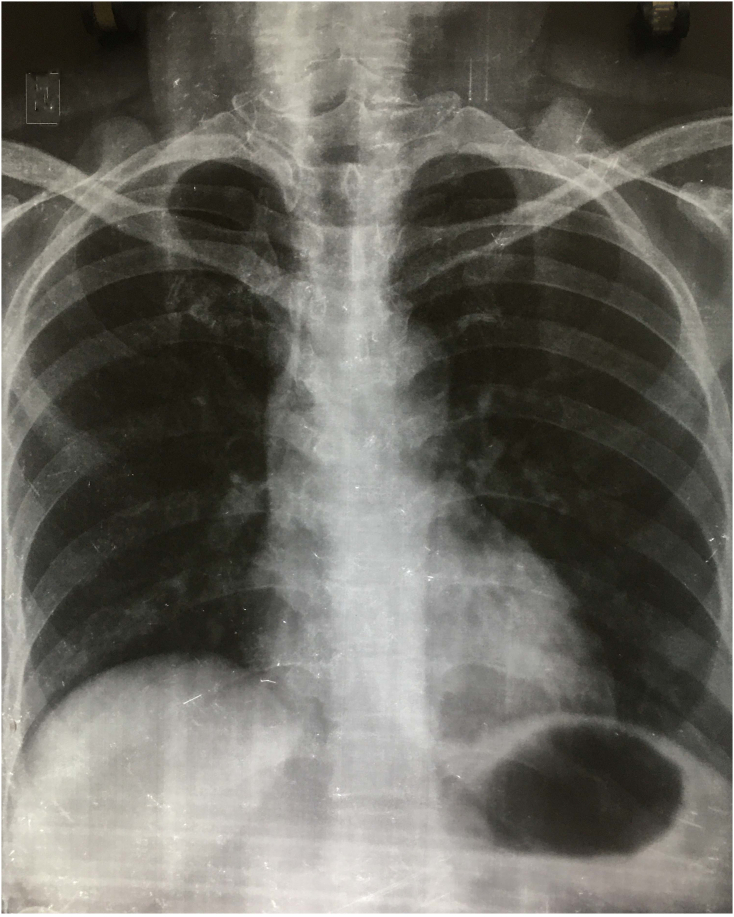
An erect X-ray chest showing no gas under right dome of diaphragm.

**Fig. 2:**
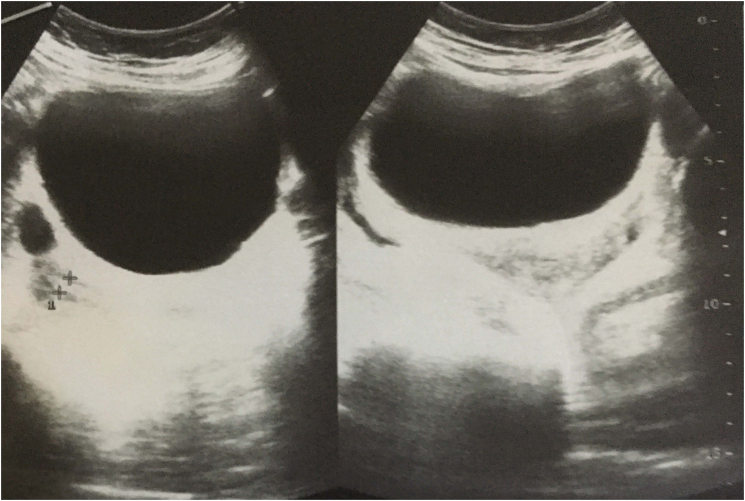
Ultrasonography showing distended gallbladder with thickening of gallbladder wall without any stone.

**Fig. 3:**
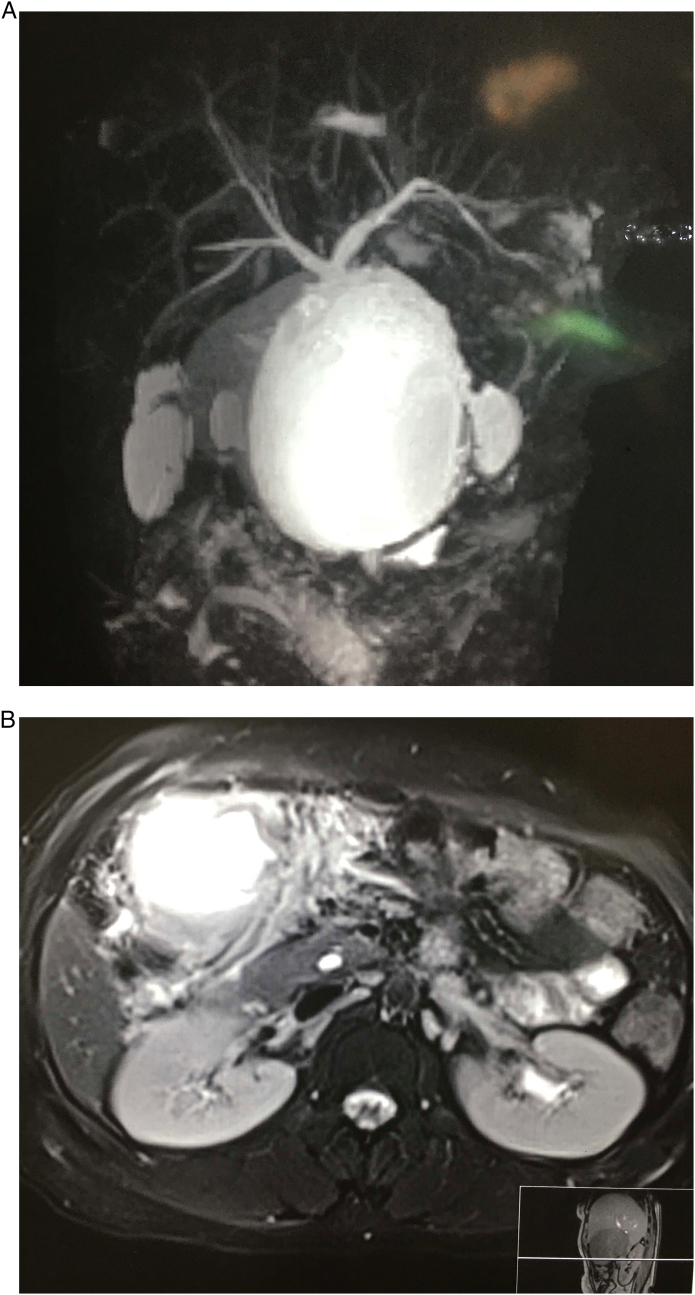
a: Magnetic resonance cholangiopancreatography (MRCP) showing a distended gallbladder with sludge, diffuse wall thickening, and contained perforation. b: Magnetic resonance cholangiopancreatography (MRCP) showing a distended gallbladder with sludge, diffuse wall thickening, and contained perforation along with inflammatory changes in the liver around the gallbladder with a mild amount of free fluid seen in the abdomen.

A 50-year-old female was admitted with a history of intermittent fever for 2 weeks. The patient also had a history of three episodes of vomiting which was non-projectile, nonbilious along with a few episodes of loose motions. There was a history of abdominal pain for 3 days. The pain was sudden onset, severe in intensity, sharp, and was associated with distension of the abdomen. There were no aggravating and relieving factors. There was no relevant history of medical and past surgical history. The patient also denied for significant family history and relevant drug history. On examination, her initial blood pressure was 110/80 mmHg, her pulse rate was 74 beats/min, her respiratory rate was 16 breaths/min, and her temperature was 37 °C (99 F). Abdominal examination showed distention and tenderness associated with guarding and rigidity in the upper abdomen. Blood investigations showed a total leucocytes count of 13,000/cmm (Neutrophil 65%, lymphocyte 30%). platelets count 100,000/l and hemoglobin of 12 mg/dl. Her liver function and renal function were within the normal limit. Erect X-ray was not revealed any free gas under the right dome of the diaphragm (Fig. 1). Abdominal ultrasonography showed distended gallbladder with thickening of gallbladder wall; there was pericholecystic fluid collection along with a collection of fluid in the upper abdomen (Fig. 2). Magnetic resonance cholangiopancreatography (MRCP) revealed a distended gallbladder with sludge, diffuse wall thickening, and contained perforation. There were inflammatory changes in the liver around the gallbladder with a mild amount of free fluid seen in the abdomen and pelvis (Fig. 3a, b). The Widal test revealed positivity for *Salmonella typhi*. With the diagnosis of type II gallbladder perforation, percutaneous ultrasonography-guided drainage was done. About 200 ml bilious drainage was found daily from the subhepatic drain. The culture of bile revealed positivity for *Salmonella typhi*. Intra-venous antibiotic (ceftriaxone and gentamicin) was administered for 14 days. Subsequently, the patient was discharged with a drain in situ. The patient was followed up two weeks later. There was a significant improvement in signs and symptoms.

**Fig. 4:**
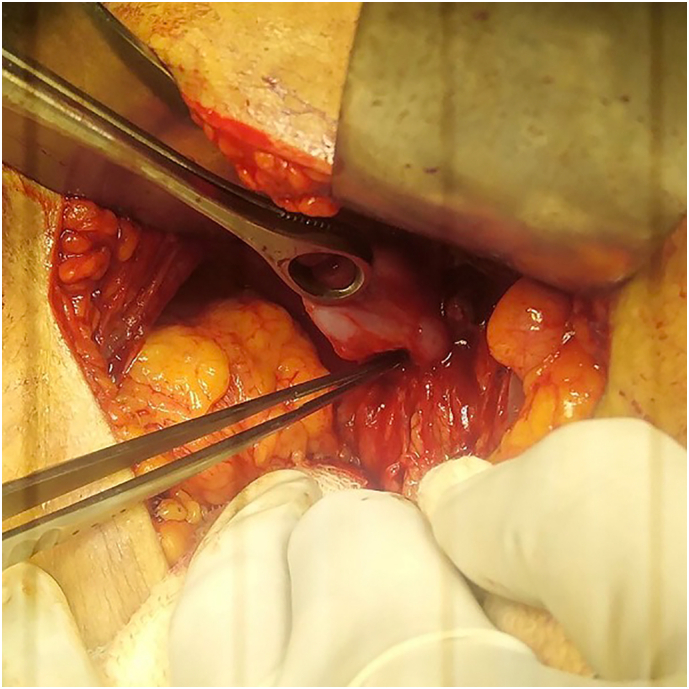
Showing perforation of gallbladder intraoperatively.

Two weeks later, surgery was planned. As our patient had presented with type II gallbladder perforation, we had expected massive adhesions and inflammation, therefore laparotomy was done. Intraoperatively, there was a dense adhesion around the gallbladder. A defect was noticed in the anteromedial aspect of the gallbladder when the peritoneal adhesion was removed (Fig. 4). The common bile duct appeared to be normal. Cholecystectomy was performed and subhepatic drain was kept after through peritoneal lavage. Postoperatively, there was a minor bile leak which was managed conservatively. The drain was removed on 7th postoperative day. Subsequently, the patient was discharged the next day.

## 4 Clinical discussion

This case report describes an uncommon presentation of enteric fever causing perforation of the gallbladder in an adult patient. Gallbladder perforation is a rare but life-threatening complication. The mortality rate of gallbladder perforation ranges from 12 to 42% [5]. Niemeier et al. had given classification for the condition as three types: type I, acute perforation into the free peritoneal cavity; type II, subacute perforation with abscess formation; and type III, chronic perforation with fistula formation between the gallbladder and another viscus [6]. The causes of gallbladder perforation have been classified as spontaneous, traumatic, and iatrogenic. The spontaneous group is further divided into an idiopathic and a secondary group. The secondary group consists of stones (acute calculous cholecystitis), acute inflammation (acalculous cholecystitis), infection (Ascaris lumbricoides, tuberculosis, and enteric fever), congenital obstruction, anticoagulant therapy, chemotherapy, diabetes, and trauma [7], [8], [9].

The most common cause of gallbladder perforation is acute calculous cholecystitis. In such cases, perforation is usually associated with obstruction leading to raised intraluminal pressure. Perforation appeared to be the result of compromised blood supply and the most common site is the distal part of the gallbladder due to its blood supply. Perforation of the gallbladder can also occur in acalculous cholecystitis [3]. Acute cholecystitis is a rare complication of acute infections like viral influenza, pneumonia, and enteric fever and gallbladder perforation is extremely rare in such cases. Perforation in such cases is likely to be the result of intense inflammation associated with acute infection and the existence of an immuno-compromised state leading to uncontrolled infection and thrombosis of the blood vessels [10], [11]. Our patient probably developed gallbladder perforation due to ischemia of the gallbladder wall because of acute intense inflammation due to acute acalculous cholecystitis.

Enteric fever is an acute systemic disease that is very common in developing countries where its association with poor sanitation and low quality of drinking water is high. Worldwide, 27 million cases and more than 200,000 deaths occur due to enteric fever [12]. Symptoms of enteric fever vary from malaise, fever, rash, abdominal pain, hepatosplenomegaly, to pancytopenia [1]. This infection commonly infects the reticuloendothelial system, liver and spleen, and predominantly bone marrow. In the tropics, enteric fever is a usual cause of intestinal perforation [13].

Early and careful diagnosis of gallbladder perforation is essential for proper treatment. Sonography is useful for diagnosing gallbladder perforation and detecting the rent in the gallbladder wall. Usually, sonography should be the first-line imaging method to assess the patients. The ultrasonographic findings of gallbladder perforation include distension (largest diameter > 3.5–4.0 cm), wall thickness (>3 mm), intracholecystic echogenic coarse debris, gallstones, and dilatation of the bile duct. As the earliest detectable signs of impending perforation could be the edema of the gallbladder wall with distention. While the ‘hole sign’ (a defect in the gallbladder wall) is the most specific finding in perforation [14]. Computerized tomography (CT) scan of the abdomen has an important role in diagnosing gallbladder perforation mainly to make the preoperative diagnosis of perforation where pericholecystic fluid is found by ultrasonography [15]. However, in some literature, it has been mentioned that plain X-ray, ultrasound, and CT have low specificity to detect gallbladder perforation [16]. The benefit of magnetic resonance cholangiopancreatography (MRCP) is to find stones in the bile ducts, dilatation bile duct, and the association of a pericholecystic fluid collection to the abdominal wall and gallbladder which information can help in surgical planning [17]. In our patient, magnetic resonance cholangiopancreatography was done which showed revealed a distended gallbladder with sludge, diffuse wall thickening, and contained perforation. There were inflammatory changes in the liver around the gallbladder with a mild amount of free fluid seen in the abdomen and pelvis.

The accurate treatment and precise timing of the surgery remain important to prevent biliary peritonitis and septic shock. Thus, early diagnosis and the initial resuscitation part is crucial with the replacement of fluid, and broad-spectrum antibiotics. In most of the type I cases, cholecystectomy and abdominal lavage are adequate to treat gallbladder perforation. Meanwhile, in type II gallbladder perforation, cholecystectomy (laparoscopic or open) is done after the infection is minimized by ultrasonography-guided percutaneous drainage [15]. However, laparoscopic cholecystectomy could be difficult in type II perforation due to dense adhesion leading to a high rate of its conversion to open cholecystectomy [18]. In addition to that, some studies have mentioned a conservative approach in selected cases [19]. Studies have also shown divided evidence regarding the indications and efficacy of open cholecystectomy over percutaneous drainage (PD) for type II perforation [5]. In one of the studies, the mortality rates following OC and PD were reported as 8.6% and 22% respectively [20]. In our case, ultrasonography-guided percutaneous drainage was performed initially and four weeks later, the definitive treatment open cholecystectomy was performed since our patient had expected massive adhesions and inflammation. Antibiotics (fluoroquinolones or ceftriaxone (1–2 g/day)) are given for 5–7 days in uncomplicated enteric fever. However, in complicated patients, antibiotics are given for 7–14 days [21]. In our case, antibiotics were given for 14 days.

In selected cases, Subtotal cholecystectomy could be the treatment of choice in difficult cholecystectomy with gallbladder perforation [18]. Nevertheless, in one of the Case series of 32 patients from Korea, the authors have described different management approaches depending upon the individual case presentation for gallbladder perforation. In two of their patients with type II gallbladder perforation, percutaneous catheter drainage followed by open cholecystectomy after approximately 2 months was performed similarly to our patient. They have also mentioned for type III gallbladder perforation, cholecystectomy can be difficult and in such cases, additional surgical procedures such as repair of the fistula along with cholecystectomy may be essential [22].

## 5 Conclusions

Gallbladder perforation is a life-threatening surgical problem that is difficult to diagnose preoperatively. The clinician should have a high index of awareness about this unusual surgical entity due to enteric fever and early diagnosis with prompt surgical intervention is necessary to improve patient outcomes.

## Funding

This research did not receive any specific grant from funding agencies in the public, commercial, or not-for-profit sectors.

## Ethical approval

Written informed consent was signed by the patient.

## Consent for publication

Written informed consent was obtained from the patient for publication of this case report and accompanying images. A copy of the written consent is available for review by the Editor-in-Chief of this journal on request.

## Credit authorship contribution statement

TRB - study concept or design, data collection, literature search, writing paper, final decision to publish.

SAK - Study concept or design, Literature search, final decision to publish.

JLJ - Study concept or design, Literature search, final decision to publish.

JKS - Study concept or design, Literature search, final decision to publish.

## Research registration number

Not applicable.

## Guarantor

Dr. Tika Ram Bhandari.

## Provenance and peer review

Not commissioned, externally peer-reviewed.

## Declaration of competing interest

All authors declare that they have no competing interests.
